# AraPPINet: An Updated Interactome for the Analysis of Hormone Signaling Crosstalk in *Arabidopsis thaliana*

**DOI:** 10.3389/fpls.2019.00870

**Published:** 2019-07-05

**Authors:** Jiawei Zhao, Yu Lei, Jianwei Hong, Cunjian Zheng, Lida Zhang

**Affiliations:** Department of Plant Science, School of Agriculture and Biology, Shanghai Jiao Tong University, Shanghai, China

**Keywords:** *Arabidopsis thaliana*, computational interactome, protein-protein interaction network, random forest, plant hormone crosstalk

## Abstract

Protein-protein interactions (PPIs) play fundamental roles in various cellular processes. Here, we present a new version of computational interactome that contains more than 345,000 predicted PPIs involving about 51.2% of the Arabidopsis proteins. Compared to the earlier version, the updated AraPPINet displays a higher accuracy in predicting protein interactions through performance evaluation with independent datasets. In addition to the experimental verifications of the previous version, the new version has been subjected to further validation test that demonstrates its ability to discover novel PPIs involved in hormone signaling pathways. Moreover, network analysis shows that many overlapping proteins are significantly involved in the interactions which mediated the crosstalk among plant hormones. The new version of AraPPINet provides a more reliable interactome which would facilitate the understanding of crosstalk among hormone signaling pathways in plants.

## Introduction

Protein-protein interactions (PPIs) play important roles in many cellular processes, including DNA replication, transcription, translation, and signal transduction. Detecting PPIs is essential for uncovering unknown functions of proteins at the molecular level and gaining insight into complex cellular networks. The importance of understanding PPIs has prompted the development of various experimental approaches such as yeast two-hybrid assay, co-immunoprecipitation and affinity chromatography for detecting PPIs (Braun et al., 2013). Although experimental techniques greatly enhanced proteomics studies, these methods remain expensive, time-consuming and labor-intensive, which even may be of uncertain reliability (Venkatesan et al., 2009).

A number of complementary computational approaches, such as gene fusion (Marcotte et al., 1999), phylogenetic profiling (Pellegrini et al., 1999), gene co-expression (Grigoriev, 2001), gene neighborhood (Rhodes et al., 2005), and interolog (Matthews et al., 2001), have been developed for prediction of PPIs based on genomic context in complete genomes. Recently, computational predictions of PPIs based on the structural context have gained much attention due to the rapid growth of protein structures (Zhang et al., 2012; Rose et al., 2015). Unlike genomic context-based methods, structure-based approaches allow for a much more detailed analysis of PPIs, which can determine the physical characteristics of the interactions and residues at the protein interface.

More recently, we have developed a hybrid computational approach by combining structural information with genomic context to predict PPIs in plants (Zhang et al., 2016). In contrast to structure-based approaches that depend on detailed information of experimental structures (Mosca et al., 2013), we calculated the characteristics of protein interfaces for interaction clues based on homology models that enabled the use of protein structural information on a genome-wide scale. By applying the hybrid method, we have constructed a computational interactome, AraPPINet, which contained more than 316,000 PPIs and showed high efficiency for discovering novel PPIs in Arabidopsis. This hybrid approach with structural information greatly increased prediction accuracy of protein interactome by largely reducing false positives on a genome-wide scale (Zhang et al., 2016; Liu et al., 2017).

Here, we present an updated version of AraPPINet that takes advantage of increasing information such as protein structure, gene expression data and functional annotation. The resulted new AraPPINet network contains over 345,000 reliably predicted PPIs with considerably increased accuracy compared to the previous version. In addition to the experimental validations reported previously, the new version was subjected to further tests that demonstrated its ability to detect PPIs involved in crosstalk of hormone signaling pathways. The updated version of AraPPINet provides a more reliable interactome which facilitates the understanding of gene function and the molecular regulation mechanisms of hormone signaling pathways in plants.

## Materials and Methods

### Preparation of Training and Testing Datasets

All experimentally determined PPIs of Arabidopsis were collected from five public databases: BioGRID (Chatr-Aryamontri et al., 2017), IntAct (Orchard et al., 2014), DIP (Salwinski et al., 2004), MINT (Licata et al., 2012), and BIND (Isserlin et al., 2011). The PPIs derived from low-throughout experiment or the interactions supported by at least two independent high-throughput experiments were extracted as positive reference dataset in May 5, 2017 (Table 1). Protein pairs without any experimental evidence for interactions were randomly chosen to generate the negative reference dataset for model training. To overcome the problem of training set imbalance, we oversampled the positive class by synthetically generating additional samples with the SMOTE method (Fernandez et al., 2018).

**Table 1:** The gold standard PPI data from various databases.

Database	BioGRID	IntAct	DIP	BIND	MINT	Unique PPIs in total
BioGRID	8,953	6,603	95	107	22	8,953
IntAct	–	6,861	48	128	30	6,861
DIP	–	–	146	3	0	146
BIND	–	–	–	153	1	153
MINT	–	–	–	–	30	30
All	–	–	–	–	–	9,260


Two independent experimentally determined datasets, including high-throughput PPIs supported by only one publication and the whole newly reported PPIs collected after May 5, 2017, were used to evaluate the performance of different computational prediction methods.

### Calculation of Structure-Based Feature

The homology models of Arabidopsis proteins were constructed by ModPipe (Pieper et al., 2014). A representative homology structure with the highest ModPipe quality score was selected for each protein according to the previously described criteria (Zhang et al., 2016; Liu et al., 2017). A total of 25,557 homology models were generated for Arabidopsis.

The structural data of over 205,000 protein complexes was collected from both the protein data bank (PDB) (Rose et al., 2015) and proteins, interfaces, structures, and assemblies (PISA) databases (Xu et al., 2008). A total of 378,000 chain–chain binary interfaces of protein complexes were generated by PIBASE with an interatomic distance cut-off of 6.05 Å (Davis and Sali, 2005). Approximately 10 billion structural comparisons were created by aligning the protein homology models to the chains of complexes using TM-Align (Zhang and Skolnick, 2005). With a normalized TM score cut-off of 0.4 for structural similarity, over 88 million homology model-chain alignments were remained for building interaction models.

Four structural features including structural similarity (TM-score), structural distance (RMSD), preserved interface size and fraction of the preserved interface were calculated from structural superposition of homology models and complexes. The detailed method of structural features calculated for each interaction model refers to the previous paper (Zhang et al., 2016).

### Calculation of Genomic-Based Feature

Gene co-expression analysis was performed based on 594 Arabidopsis RNA-Seq data collected from SRA database in NCBI. The cleaned reads without adaptors and low-quality ones were then mapped to Arabidopsis genome sequences by using Tophat2 (Trapnell et al., 2012). Mapped reads were counted by using HTSeq-Counts (Anders et al., 2015). Relying on these data, reads per kilobase of transcript per million mapped reads (RPKM) (Mortazavi et al., 2008) were calculated to measure the level of gene expression. Pearson correlation coefficients were then calculated for each gene pair.

Gene functional similarity is defined as *S* = log(n/N)/log(2/N) on three independent gene ontologies (GO) (biological process, molecular function, and cellular component), where *n* represents the number of genes in the lowest GO class containing these two genes, and *N* is the total number of genes in the Arabidopsis genome annotation. The GO data used came from the ontology file (Ashburner et al., 2000). The feature based on the cellular component ontology is used to capture the cellular co-localization of proteins for PPI prediction, which could avoid the false interactions raised by spatially separated proteins (Huh et al., 2003).

The calculation method of gene phylogenetic profile similarity was the same as that of the previous network (Zhang et al., 2016). A total of 300 eukaryotic and 1,026 prokaryotic sequenced genomes were collected in the study after removing evolutionarily similar genomic data. The Arabidopsis proteins were then BLAST aligned with all of the collected protein-encoding sequences.

Interolog analysis was performed similarly to a strategy proposed by Jonsson and Bates (2006). InParanoid with default settings was used to identify the orthologs of Arabidopsis proteins with that of *Escherichia coli*, *Saccharomyces cerevisiae*, *Caenorhabditis elegans*, *Drosophila melanogaster*, *Mus musculus*, and *Homo sapiens*. The experimentally determined PPI datasets of these six model organisms used for interolog analysis were derived from the BioGRID, IntAct, DIP, and MINT databases (Supplementary Table S1).

Rosetta stone protein (gene fusion) was calculated by following the previous described method (Zhang et al., 2016).

### Prediction of Protein Interaction

Positive and negative reference sets with 11 features were used to train the random forest classifier in *R* with the setting of 500 trees (Liaw and Wiener, 2001). The whole possible protein pairs were then classified by the optimized random forest model. The predicted network was generated by Cytoscape (Cline et al., 2007).

### Evaluation of Prediction Performance

Ten-fold cross-validation method was used to evaluate the performance of the model. Training dataset was divided into 10 equal shares. Nine of them were used to train the model and the remaining one was used to test the prediction. This progress was repeated for 10 times by using the different dataset each time. The average value of these results was set as the final evaluation. The value of TP (true positive), FP (false positive), TN (true negative) and FN (false negative) were counted. True positive rate (TPR) or recall = TP/(TP + FN), false positive rate (FPR) = FP/(FP + TN) and precision = TP/(TP + FP) were calculated to measure the performance of the model.

Precision-recall (PR) curves were produced from the Random Forest classifier testing progress in order to compare the prediction performance of the updated AraPPINet with the earlier version (Zhang et al., 2016). Additionally, the performance of the updated AraPPINet was compared with that of the previous version and three other available PPI prediction methods: AtPID (Cui et al., 2008), AtPIN (Brandão et al., 2009) and PAIR (Lin et al., 2011) based on the test datasets. The latest versions of the three methods (AtPID V5.03, AtPIN V9, and PAIR V3) were used to predict PPIs and compare the values of TPRs and F-measures.

### Significance Analysis of Pathway Interaction

Proteins interacted with the core proteins of hormone signaling pathway were predicted by the updated AraPPINet. A Fisher’s exact test was performed to test the significance of overlapping interacting proteins between two hormone signaling pathways based on the following 2 × 2 table,

**Table:** 

	Hormone 1	Not Hormone 1

Hormone 2	X_ 12_	X_2_–X_12_
Not Hormone 2	X_1_–X_12_	13,929-X_1_–X_2_+X_12_


where X_1_ and X_2_ are the numbers of interacting proteins in hormone signaling pathway 1 and 2, respectively; X_12_ is the number of overlapping proteins between the two hormone signaling pathways. A total of 13,929 proteins were contained in the updated AraPPINet network. *P*-value obtained from reference data (using function fisher test in *R*) with the level less than 0.001 indicates the significance of overlapping interacting proteins between two hormone signaling pathways.

### Function Analysis of Hormone-Interacting Proteins

Functional enrichment of hormone-interacting proteins was gained from database for annotation, visualization, and integrated discovery (DAVID) (Huang da et al., 2009) by comparing the genes with their FDR values.

### Enrichment Analysis of Interactions Between Hormone Signaling Pathways

To estimate the possibility of core components linked to proteins in another hormone signaling pathway, an edge-shuffling method was used to generate 1000 randomized networks with the same degree of each node as that in the protein interaction network. The number of interactions of core components between two hormone signaling pathways was then counted in the original network and the randomized networks. The fold enrichment of interactions was calculated by dividing the observed links in original network by that in the randomized networks.

## Results

### Updating PPI Networks in Arabidopsis

**FIGURE 1:**
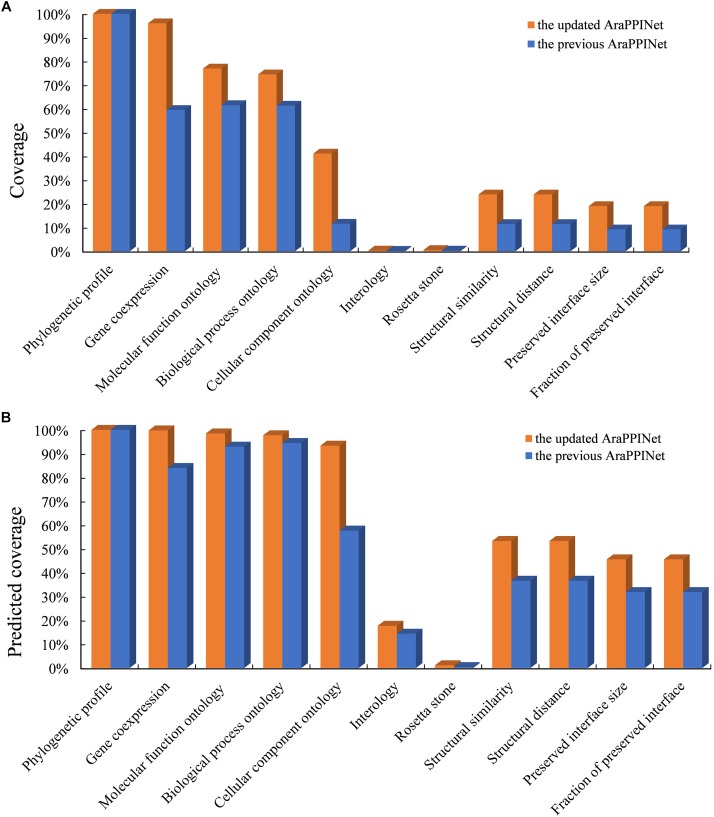
Comparison of feature coverage in the updated and the previous versions. **(A)** Data coverage of all protein pairs with the listed features in the updated and the previous datasets. The coverage represents a ratio of the number of protein pairs with available information in each feature divided by the number of whole possible protein pairs. **(B)** Data coverage of the predicted PPIs with the given features in the two AraPPINet versions. The coverage represents a ratio of the number of predicted PPIs with available information in each feature divided by the number of all predicted interactions.

We have updated the Arabidopsis PPI networks inferred from both structural information and genomic contexts. Currently, the genomic features were improved by new data, such as RNA sequencing data, which greatly increased the data coverage of gene co-expression from 59.51 to 96.02%. Moreover, 23.94% of all possible protein pairs were supported by structural information, which is much higher than the previous version (11.48%). The higher data coverage would improve the accuracy of model for discovering PPIs (Figure 1A).

The training datasets with different ratios of positive to negative samples were used to train the model. When the model was trained by a dataset consisting of positive and negative samples of equal size (1:1), the predicted model presented a relatively high TPR of 70.62%. Although the accuracy of the trained model had been relatively well, a higher FPR of 14.97% would result in a large number of FP interactions on a genome-scale (Table 2). When more negative examples were added to the training dataset, the enlarged negative dataset effectively decreased the FPR, but meanwhile the imbalance in training dataset significantly compromised the TPR (Table 2). In order to reduce the imbalance level, synthetic minority oversampling technique (SMOTE) was applied to synthetically generate additional samples for the positive class (Fernandez et al., 2018). The model performance became better as the ratio of positive training samples increased. When the ratio of positive to negative samples was optimized to 1:40, the generated model showed a relatively high TPR of 49.74%, while the FPR (0.095%) remained at the low level of less than 0.1% (Table 2). This level of FPR was expected due to the use of random protein pairs as the negative dataset in model training (Arabidopsis Interactome Mapping Consortium, 2011).

**Table 2:** Performance comparisons of PPI predictions with and without SMOTE technique.

Positive to negative ratio	SMOTE or not	TPR on training set (%)	FPR on training set (%)	TPR on test set (%)	FPR on test set (%)
1:1 (9260:9260)	No	70.62	14.97	64.01	14.07
1:10 (9260:92600)	No	40.05	0.839	22.36	0.753
1:50 (9260:463000)	No	27.51	0.114	11.51	0.098
1:60 (9260:555600)	No	26.34	0.092	10.76	0.086
1:80 (9260:740800)	No	24.64	0.061	9.64	0.065
1:100 (9260:926000)	No	23.19	0.047	9.01	0.055
1:10 (92600:1389000)	SMOTE	84.51	0.405	18.77	0.179
1:30 (46300:1389000)	SMOTE	63.33	0.103	14.47	0.101
**1:40 (34725:1389000)**	**SMOTE**	**49.74**	**0.095**	**14.20**	**0.095**
1:50 (27780:1389000)	SMOTE	47.94	0.071	13.78	0.072
1:75 (18520:1389000)	SMOTE	28.29	0.047	12.29	0.057


*The bold values mean the optimized positive to negative ratio of training set with a relatively high TPR and a low level of FPR.*

By applying the optimized PPI prediction model, we constructed a new version of Arabidopsis PPI network. The updated AraPPINet contained 345,006 reliable PPI predictions involving 13,929 (51.2%) proteins, about 8.9% more interactions and 10.8% more proteins than the previous version. Over half of the predicted PPIs were supported by structural information, which is much higher than that of the earlier version (Figure 1B). Similar to the previous version, the new protein interaction network also exhibited free-scale traits, and its high-clustering coefficient values revealed that the topological structure was highly modular (Supplementary Figure S1).

### Prediction Accuracy With Respect to the Previous Methods

**FIGURE 2:**
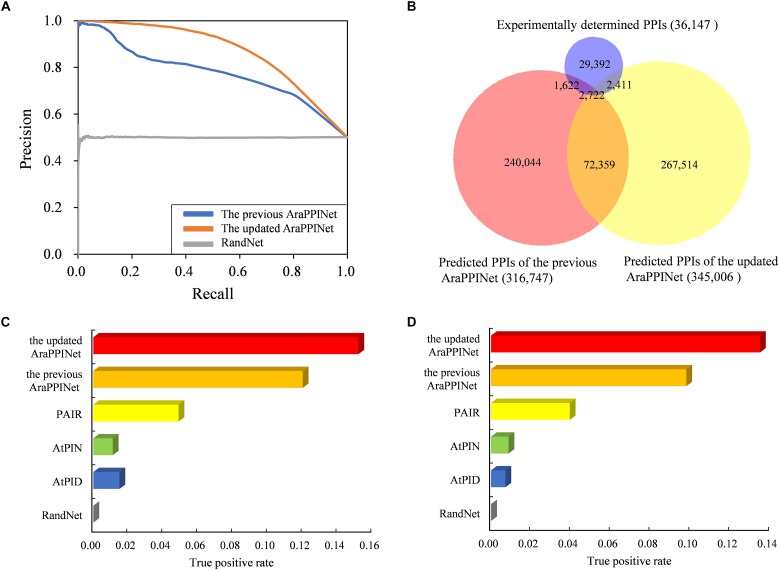
Performance comparison of the updated AraPPINet with other computational methods. **(A)** PR curves of the updated AraPPINet and the previous version on test dataset. **(B)** Venn diagram of predicted PPIs overlapping with the experimentally determined protein interactions. **(C)** Comparison of the updated AraPPINet with other methods based on the high-throughput dataset. **(D)** Comparison of the updated AraPPINet with other methods based on the newly reported interactions.

We assessed the performance of the current method with respect to the previous version. Firstly, a 10-fold cross-validation was performed using the training datasets. Performance evaluation showed that the TPR of the new method reached 49.74%, while the FPR remained at the low level of 0.095%. PR curves based on test dataset as well as on training dataset showed that the new method had a higher area under the curve (AUC) value than the previous version (Figure 2A and Supplementary Figure S2). These results suggested that the updated AraPPINet network was much more efficiency and accuracy than the previous version. Moreover, among the 36,147 experimentally determined PPIs without training positive interactions, a total of 5,133 PPIs were predicted by the updated model, and much higher than the previous version (Figure 2B).

Furthermore, we employed two independent datasets as the benchmarks to compare the performance of the different computational methods: in discovering PPIs. One is the dataset derived from the high-throughput PPIs supported by only one publication, and another one is the dataset comprising all PPIs released after May 5, 2017. Among the 14,815 high-throughput PPIs, 2,252 (15.2%) protein interactions could be successfully predicted by the updated AraPPINet, which showed better performance than the previous version and three other methods: AtPID, AtPIN, and PAIR (Figure 2C). Moreover, we evaluated the abilities of these different methods to predict novel PPIs with the newly reported dataset. Of these 21,332 newly reported PPIs, 2,880 (13.5%) protein interactions were successfully recognized by the updated model, which significantly overperformed the four other methods (Figure 2D). We also compared F-measure of different prediction methods using the test datasets and the updated AraPPINet showed great improvement over other PPI prediction methods based on the F1 score (Tables 3, 4). These results suggested that the new model could effectively increase the accuracy of the predicted protein interactome.

**Table 3:** F-measure comparison of different prediction methods based on high-throughput dataset.

Method	Positive PPIs	All predicted PPIs	Precision	Recall	F1 score
RandNet	123	345,006	0.0004	0.0083	0.0007
AtPID	222	24,418	0.0091	0.0150	0.0113
AtPIN	165	87,936	0.0019	0.0111	0.0032
PAIR	724	137,837	0.0053	0.0489	0.0095
The previous AraPPINet	1,779	316,747	0.0056	0.1201	0.0107
The updated AraPPINet	2,252	345,006	0.0065	0.152	0.0125


**Table 4:** F-measure comparison of different prediction methods based on newly released dataset.

Method	Positive PPIs	All predicted PPIs	Precision	Recall	F1 score
RandNet	124	345,006	0.0004	0.0058	0.0007
AtPID	154	24,418	0.0063	0.0072	0.0067
AtPIN	187	87,936	0.0021	0.0088	0.0034
PAIR	843	137,837	0.0061	0.0395	0.0106
The previous AraPPINet	2,091	316,747	0.0066	0.098	0.0124
The updated AraPPINet	2,880	345,006	0.0083	0.135	0.0157


### Inference of Major Hormone Signaling Networks

**FIGURE 3:**
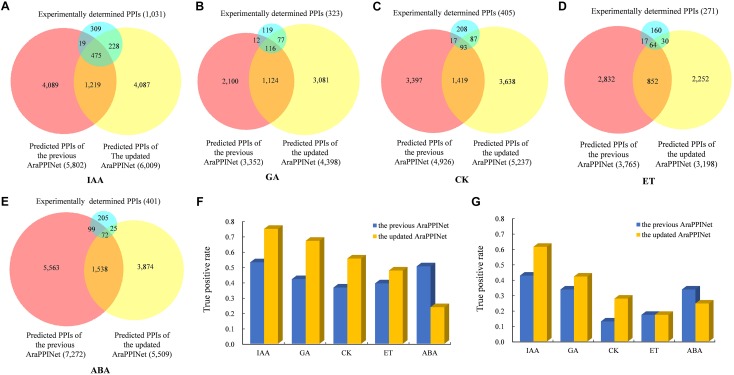
Performance comparison of the two AraPPINet versions for predicting hormone signaling networks. **(A–E)** Venn diagram of predicted PPIs involved in different hormone signaling overlapping with the experimentally determined PPIs **(F)** Comparison of the two AraPPINet versions for predicting PPIs involved in hormone signaling based on the high-throughput dataset. **(G)** Comparison of the two AraPPINet versions for predicting PPIs involved in hormone signaling based on the newly reported interactions.

Plant hormones play important roles in regulating plant growth, development, and responses to the environment. Although many components of hormone signaling pathways are well known, the signaling networks consisting of interactions between core components and their related proteins are still largely unclear. By inferring from the updated version of AraPPINet, we constructed the signaling networks of five hormones including gibberellin (GA), auxin (IAA), cytokinin (CK), ethylene (ET), and abscisic acid (ABA). Compared to the previous version, the new version of AraPPINet could predict more PPIs involved in the hormone signaling networks of GA, IAA, and CK in the new protein interaction network (Figure 3A–E). For example, a total of 4,398 interactions were predicted to be related to GA signaling in the new version, while 3,352 PPIs were identified in the previous version. Moreover, the accuracy of predicted PPIs in hormone signaling networks was evaluated by two independent datasets of experimentally verified interactions. For IAA signaling, a total of 518 predicted PPIs were identified from high-throughput experiments, of which 388 (75%) protein pairs could be successfully predicted by the new network, that was much more accuracy than the previous version (53%) (Figure 3F). Among 513 newly reported PPIs involved in IAA signaling, 315 (61%) PPIs were predicted by the new network, while 219 (43%) interactions were recognized by the previous network (Figure 3G). Furthermore, we also evaluated the accuracy of the predicted PPIs associated with other hormone signaling pathways. As shown in Figure 3F,G, apparently the new network shows great improvement in PPI prediction of GA, CK, and ET signaling pathways over the previous version on both two test datasets. All these results suggested that the new version of AraPPINet was more powerful to discover novel PPIs involved in plant hormone signaling pathways.

### Interplay of Hormone Signaling Pathways

**FIGURE 4:**
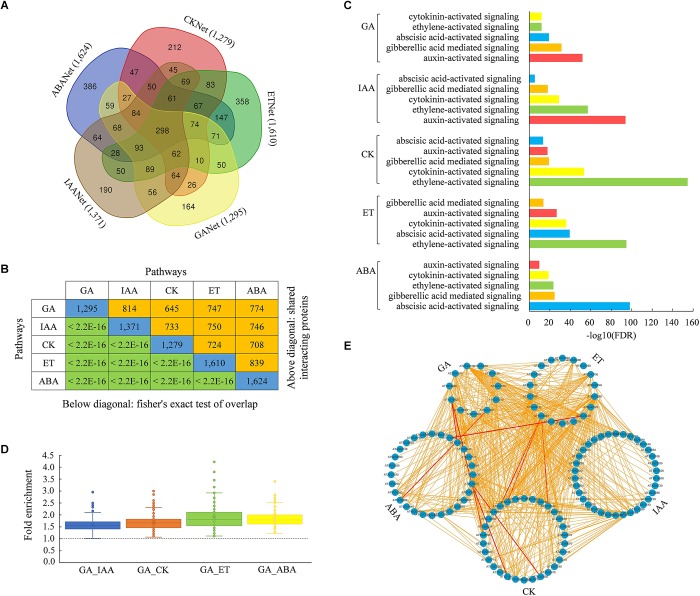
Inference of hormone signaling crosstalk from the updated AraPPINet. **(A)** Venn diagram of hormone signaling interacting proteins inferred from the updated network. **(B)** Overlapping interacting proteins of five hormone signaling pathways. Numbers on the diagonal line represent total number of proteins interacting with each hormone signaling. The number of common interacting proteins between any two hormone signaling pathways is indicated above the diagonal. *P*-value of the overlap obtained from fisher’s exact test is presented below the diagonal line. **(C)** Enriched GO functional categories of proteins interacting with each hormone signaling pathway. **(D)** Enrichment folds of interactions connected core components of GA with that in other hormone signaling pathways. **(E)** Interactions among core components in five hormone signaling pathways. All predicted connections among core proteins are colored in yellow, and experimentally demonstrated interactions are colored in red.

Plant hormone signaling pathways are often interconnected with each other in the regulation of diverse biological processes. To identify common proteins involved in different hormone signaling, we analyzed candidate proteins that were interacted with the core components of GA, IAA, CK, ET, and ABA signaling pathways and compared these interacting proteins inferred from the new AraPPINet. Remarkably, many interacting proteins were overlapped in two or more hormone signaling networks (Figure 4A). According to the Fisher’s exact test analysis, we found that the level of overlap between hormone signaling networks was much greater than expected at random, indicating that all five hormone signaling pathways significantly interact with each other (Figure 4B). Moreover, a subset of well-represented GO terms was used to identify the functional trends of the interacting proteins in each hormone signaling. Proteins interacting with each hormone signaling were significantly involved in the regulation of other four hormone signaling pathways (Figure 4C and Supplementary Table S2), suggesting that many of these proteins play important roles in the regulation of more than one hormone signaling pathway.

Among these interacting proteins, some are core components in hormone signaling pathways. We found that many core proteins could also directly interact with each other in different hormone signaling pathways. Enrichment analysis of protein interactions between GA and another pathway revealed that GA pathway had a high possibility in connecting with different hormone signaling pathways through the interactions of the core components (Figure 4D and Supplementary Table S3). The network consisting of all predicted links among the core components of five signaling pathways is shown in Figure 4E. These results suggested that the new interactome had a high possibility in discovering connections among core proteins in different hormone signaling pathways.

**FIGURE 5:**
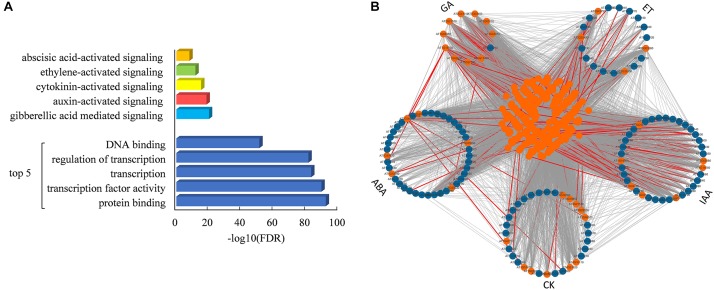
Functional enrichment and interaction network of overlapping proteins of five hormone signaling pathways. **(A)** Enriched GO functional categories of 298 overlapping proteins of five hormone signaling pathways. **(B)** Interaction network of overlapping proteins and core components in hormone signaling pathways. Orange and blue nodes represent overlapping proteins and core components, respectively. All predicted links among these proteins are colored in gray and experimentally demonstrated edges are colored in red.

It is interesting that 298 proteins were predicted to interact with all five hormone signaling pathways (Figure 5A). Functional enrichment analysis showed that these overlapping proteins were significantly involved in the protein binding and regulation of transcription, as well as the regulation of five hormone signaling pathways (Figure 5A and Supplementary Table S4). As shown in Figure 5B, a total of 22,292 interactions are found among these overlapping proteins and core components of five hormone signaling pathways, of which 224 interactions have been proved by experimental assays. All of these results indicated that these overlapping proteins might play important roles in connecting five hormone signaling pathways in plants.

## Discussion

In this study, we present a new version of AraPPINet, which contains over 345,000 reliably predicted interactions, almost 30,000 PPIs more than the previous version. Most of the increase of PPI predictions is due to the availability of new structural information and new high-throughput genomic data (Figure 1). Among the used data sources, structural distance, and biological process ontology are the key structural and genomic clues affecting the performance of the new method, respectively. It should be noted that proteins cannot interact with each other *in vivo* if they are spatially separated in a cell. To avoid the false interactions raised by spatially separated proteins, a feature based on the cellular component ontology has been used to capture the cellular co-localization of proteins for PPI prediction (Ashburner et al., 2000; Huh et al., 2003). These various interaction clues have been combined to make reliable PPI predictions on a genome-wide scale (Zhang et al., 2016).

In addition to the use of new data, the predictability of new AraPPINet is also benefited from the reduced effects of class imbalance by using SMOTE technique, which can improve the prediction of the positive interactions (Fernandez et al., 2018). Moreover, we used the optimized ratio of positive to negative sample in training dataset to prevent FPs from a large amount of non-interacting protein pairs. This strategy has been shown to effectively increase the accuracy of PPI prediction by reducing the FPR to the expected level (Zhang et al., 2016; Liu et al., 2017). Performance comparisons showed our optimized model significantly performed better than the other developed methods, which could be able to make reliable predictions on a genome-wide scale.

Different hormone signaling pathways can interact with each other to regulate plant growth and development. The extensive crosstalk among individual hormone pathways leads to the formation of complex signaling networks (Murphy, 2015). The evaluations with independent datasets suggested that the new AraPPINet provides a valuable resource for identifying protein interactions involved in hormone signaling networks of plants. Indeed, the performance of the new network appears comparable to that of experimental databases as measured by TPR, and its coverage is far more extensive than that of the previous network. Network analysis showed different hormone signaling pathways extensively interact with each other in the updated AraPPINet. Interestingly, we found approximately 300 overlapping proteins involved in the interplay of all five hormone signaling pathways. For example, JAZ1 (AT1G19180), a key repressor of JA signaling, was predicted to interact with the core components in other four hormone signaling pathways, of which interactions between JAZ1 and RGL1 (AT1G66350), and JAZ1 and EIL1(AT2G27050) have been shown to modulate JA and GA, and JA and ET signaling pathways, respectively (Hou et al., 2010; Zhu et al., 2011). The increasing evidences suggested these common proteins would serve as central regulators that mediate the crosstalk of various hormones. The new version off computational interactome greatly expanded the known interactions involved in hormone signaling networks, which would facilitate the understanding of hormone crosstalk in plants.

## Author Contributions

JZ and LZ designed the project, carried out the model training and computational validation, and wrote the manuscript. YL performed the homology modeling. JH performed the bioinformatic analyses. CZ made substantial contributions to data collection. All authors approved the final version of the manuscript.

## Conflict of Interest Statement

The authors declare that the research was conducted in the absence of any commercial or financial relationships that could be construed as a potential conflict of interest.

## Supplementary Material

FIGURE S1 Topological properties of the updated and the previous AraPPINet networks. **(A)** Degree distribution of the node proteins. **(B)** Average clustering coefficient of proteins with the same degree.

FIGURE S2 PR curves of the updated AraPPINet and the previous version on training dataset.

TABLE S1 Experimentally determined PPIs of six model organisms.

TABLE S2 Enriched GO functional categories of proteins interacted with hormone signaling pathways.

TABLE S3 List of core proteins significantly interacting with GA signaling pathway.

TABLE S4 Enriched GO functional categories of overlapping proteins of five hormone signaling pathways.
