# Supplementary figures and images for: AraPPINet: An Updated Interactome for the Analysis of Hormone Signaling Crosstalk in Arabidopsis thaliana
Source: Front Plant Sci. 2019 Jul 5;10:870. doi: 10.3389/fpls.2019.00870 (PMC6625390; doi:10.3389/fpls.2019.00870)

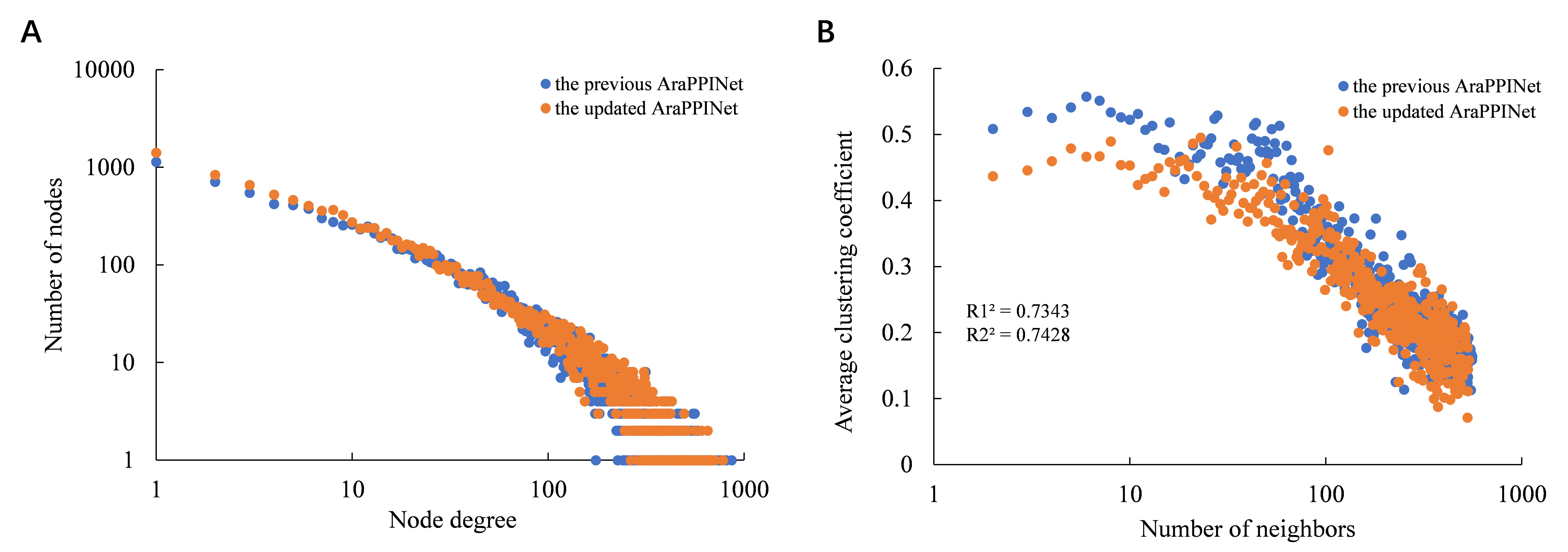

Supplement: FIGURE S1 — Topological properties of the updated and the previous AraPPINet networks. (A) Degree distribution of the node proteins. (B) Average clustering coefficient of proteins with the same degree. [file Image_1.JPEG]

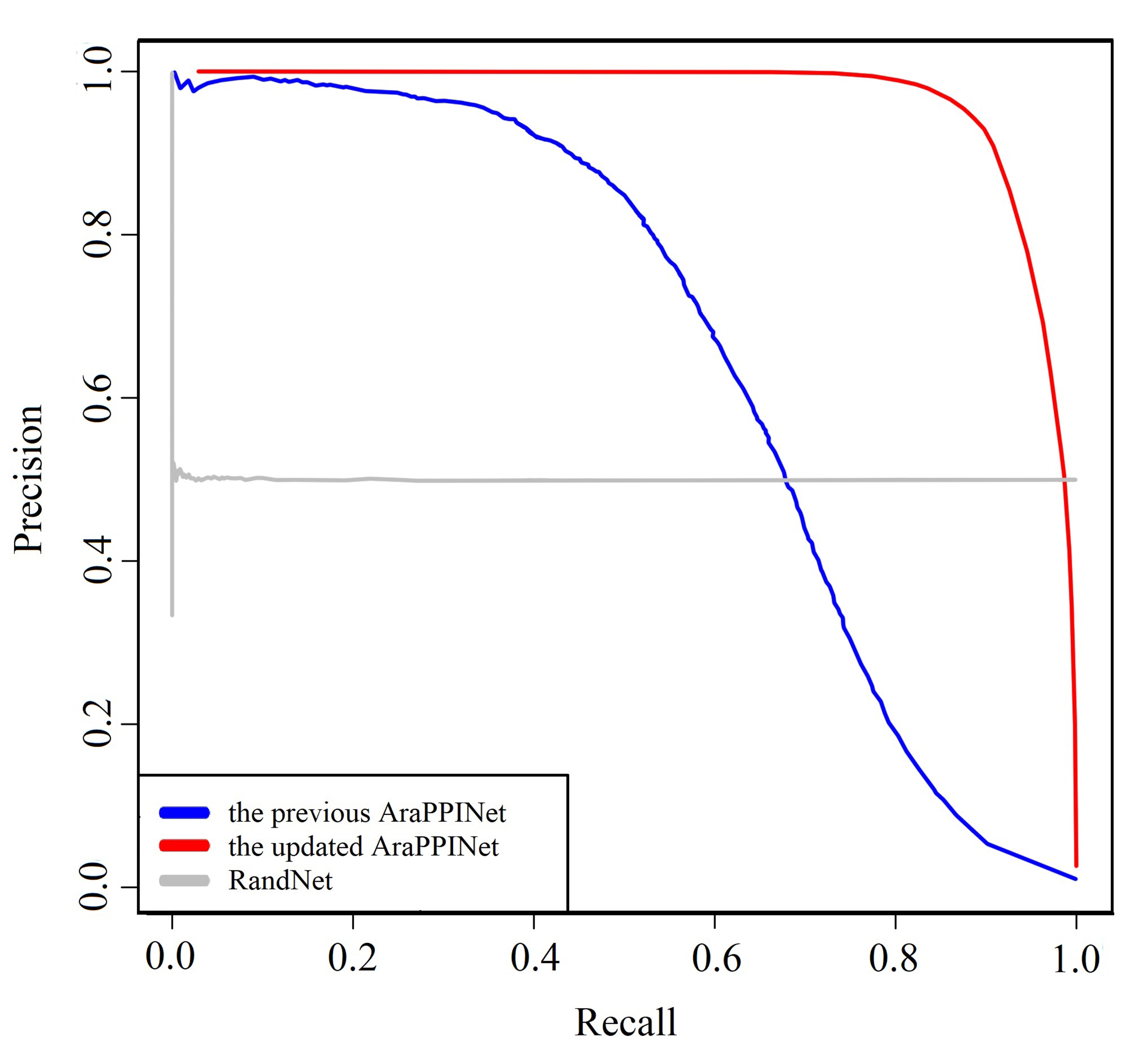

Supplement: FIGURE S2 — PR curves of the updated AraPPINet and the previous version on training dataset. [file Image_2.JPEG]
